# Iron-doped ZnO as a support for Pt-based catalysts to improve activity and stability: enhancement of metal–support interaction by the doping effect†

**DOI:** 10.1039/c8ra03664k

**Published:** 2018-06-12

**Authors:** Si Bui Trung Tran, Han Seul Choi, Sun Young Oh, Song Yi Moon, Jeong Young Park

**Affiliations:** Centre for Nanomaterials and Chemical Reactions, Institute of Basic Science (IBS) Daejeon 305-701 Republic of Korea jeongypark@kaist.ac.kr; Graduate School of EEWS and Department of Chemistry, Korea Advanced Institute of Science and Technology (KAIST) Daejeon 305-701 Republic of Korea

## Abstract

In heterogeneous catalysis, the role of the interface between a metal and a metal oxide in deciding catalytic performance has remained a long-standing question. Out of many molecular-scale factors that affect the properties of metal–oxide interfaces, doping or impurities in the oxides can result in excess charge carriers or oxygen vacancies on the oxides, which lead to a change in catalytic activity. For a model system with a tunable dopant, we employed Pt nanoparticles with Fe doping. We synthesized a series of Fe-doped ZnO with different Fe loadings (*i.e.*, 0, 1, and 4%) using the co-precipitation method, and then deposited Pt nanoparticles onto these supports. The Pt-based catalysts were employed to investigate the effect of the dopant to promote the catalytic performance for the CO oxidation reaction. The 4% Fe loading sample showed the highest catalytic activity among the catalysts, with a turnover frequency of 5.37 s^−1^ at 126 °C. The dopant was found to enhance the interaction between the Pt nanoparticles and the catalyst support, including the prevention of metal sintering, which resulted in an improvement of catalytic activity.

## Abstract 2

We deposited Pt nanoparticles on a series of Fe-doped ZnO with different Fe loadings to study the effect of dopant on the catalytic performance for the CO oxidation reaction.

## Graphical abstract

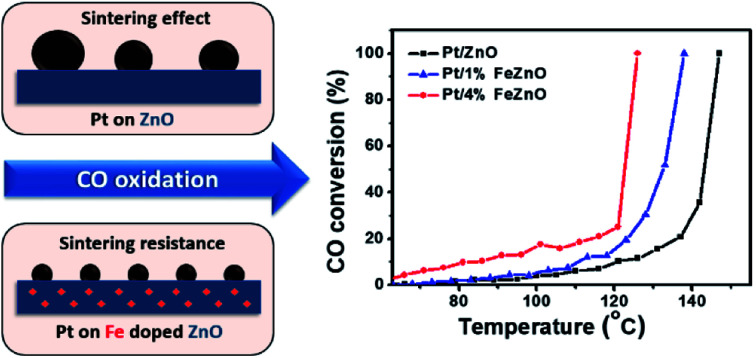

## 1 Introduction

In heterogeneous catalysis, the interface between a metal and a metal oxide plays a crucial role in deciding catalytic performance. Schwab *et al.* showed an increase in catalytic activity for methanol oxidation on an Ag/ZnO catalyst based on the existence of an interaction between Ag and ZnO.^1^ Later, the classical strong metal–support interaction (SMSI) effect, discovered by Tauster *et. al.* on TiO_2_ supported on a group VIII metal,^2^ involved the transfer of electrons from the catalyst support to the metal after H_2_ treatment at high temperature. Since then, the SMSI effect has been studied and extended to other phenomena related to the interaction between a metal and a metal oxide, which determines both the catalytic activity and selectivity.^3–8^ The electronic factor that originates from charge transfer at the interface between the metal and the oxide support, and the geometric factor that results from a change in the metal and/or metal oxide morphological structure are used to rationalize the SMSI effect.^9–12^ With respect to geometric factors, when the interaction between the metal and metal oxide is very weak, metal nanoparticles (NPs) have a tendency to agglomerate to form larger NPs on the metal oxide surface, known as the sintering effect. However, when this interaction is strong, encapsulation of the metal by the metal oxide is often observed, thus maximizing the interfacial area. As a nanostructure of metal particles is vitally important in catalysis, the design of novel catalysts with the desired metal–support interaction is beneficial for improving catalytic performance.

Since metal oxides have been employed as catalyst supports for a variety of industrial processes,^13–15^ there is great demand for optimizing their properties to improve catalytic performance. Among the different methods to achieve this goal, the substitution of a minor fraction of the cations in the host oxide lattice with external metal ions, referred to as the doping process, has gained much attention.^16–18^ This process can modify the electronic structure as well as the physical structure (*e.g.*, the crystallinity or surface area) of the host oxide. As a result, when doped metal oxides are used as catalyst supports, this modification inevitably leads to a change in the interaction between the doped metal oxide and the metal NPs grown on them. This strategy has been utilized for the development of novel doped catalysts that are able to control catalytic performance.^19,20^ For instance, Schumann *et al.* investigated the dopant effect of Al, Ga, and Mg on the support in Cu/ZnO catalysts for methanol synthesis and the reverse water–gas shift reaction.^21^ They found that the dopants Al^3+^ and Ga^3+^ improved the electronic contribution to the reverse water–gas shift reaction and promoted structural contributions in methanol synthesis, while Mg^2+^ had no beneficial influence on the activity. Peng *et al.* introduced Mg^2+^ into ZnO to improve the catalytic stability for CO oxidation coupling to dimethyl oxalate.^22^ A small portion of the Mg^2+^ was found to be incorporated into the lattice of the ZnO support, which led to a SMSI caused by electron transfer from the ZnO to the Pd NPs. Apparently, a small amount of dopant can contribute to a change in the catalytic outcome; hence, understanding the influence of dopants on catalytic performance would be interesting and desirable to research further.

The semiconductor ZnO has been considered as a host oxide for doping different metal ions to alter their physical and chemical properties.^23,24^ ZnO, which belongs to the group of non-reducible metal oxides because of their intrinsic resistance to any change in oxidation state, is highly stoichiometric, stable, and chemically inert. Therefore, when it is doped with other metal ions, any change in catalytic performance should be attributed to the dopant. Herein, we employed Fe-doped ZnO with different Fe loadings as catalyst supports for the deposition of Pt NPs. The fabricated Pt-based catalysts were tested using CO oxidation as the probe reaction to study the influence of the metal dopant on catalytic performance. We found that a small amount of Fe dopant enhanced the catalytic activity, which was attributed to an enhancement of the interaction between the Pt NPs and the catalyst support.

## 2 Experimental

### 2.1 Preparation of Fe-Doped ZnO and Pt/*x*%FeZnO catalysts

Fe-doped ZnO samples were synthesized using the co-precipitation method with zinc nitrate as the starting material, ferric nitrate as the doping source, and urea as the precipitator. Aqueous solutions with calculated amounts of metal nitrates (*i.e.*, Zn(NO_3_)_2_·6H_2_O and Fe(NO_3_)_3_·9H_2_O) were prepared separately. They were then mixed and stirred for 2 hours. The urea solution was then slowly added to the above mixture. The final solution was thoroughly stirred for 2 hours, and then heated to and kept at 90 °C for 1 hour to yield precipitates. The precipitates were collected by centrifugation, washed with distilled water and ethanol, and then dried in an oven at 110 °C overnight. The dried precipitates were calcined at 600 °C for 5 hours to obtain Fe-doped ZnO powders. ZnO was synthesized using the same procedure without adding the Fe precursor. All the samples are denoted as *x*%FeZnO, where *x* (*i.e.*, 0, 1, and 4) is the percentage of doped Fe.

Platinum-based catalysts were prepared by the deposition–precipitation method using H_2_PtCl_6_ as the precursor. In a typical preparation, an aqueous solution was prepared containing 600 mg of urea in 40 ml of H_2_O, followed by the addition of 900 mg of the *x*%FeZnO sample. The mixture was stirred for 1 hour, and then a solution of 85 mg H_2_PtCl_6_ in 30 ml H_2_O was added. The mixture was then stirred for 3 hours and subsequently heated to 90 °C and aged at that temperature for 2 hours. The mixture was cooled down to room temperature and a solution of 60 mg NaBH_4_ dissolved in 10 ml of H_2_O was then added dropwise. The reaction was continued for 1 hour with stirring, and the black precipitate was centrifuged, washed 5 times with water, and dried in an oven overnight at 110 °C. The prepared samples were denoted as Pt/*x*%FeZnO, where *x* (*i.e.*, 0, 1, and 4) is the percentage of doped Fe.

### 2.2 Characterization and catalytic reaction

The size and morphology of the synthesized catalysts were assessed by transmission electron microscopy (TEM, Tecnai F30 microscopy operated at 300 kV) accompanied with energy dispersive X-ray spectroscopy (EDS). X-ray diffraction (XRD) patterns of the samples were recorded using a D/MAX-2500 (Rigaku; at 40 kV and 300 mA) that scanned 2*θ* values between 25° to 85°. A VG Scientific Sigma Probe X-ray photoelectron spectroscopy (XPS) system equipped with an Al Kα X-ray source (1486.3 eV) under ultra-high vacuum at 10^−10^ Torr was used to analyse the oxidation states of the Pt. Ultraviolet-visible (UV-vis) spectroscopy was carried out on a Lambda 1050 (Perkin Elmer) equipped with 10 mm quartz cells at room temperature. The Brunauer–Emmett–Teller surface area and pore structure of the synthesized catalysts were determined using the N_2_ adsorption method (Micromeritics Tristar II 3020 V1.03 Analyzer). The amount of Fe and Pt loading was measured by inductively coupled plasma optical emission spectroscopy (ICP-OES) using a Thermal Scientific iCAP 6300. Metal dispersion on the samples was measured using CO pulse chemisorption (BELCAT-B; BEL Japan Inc.) with a stoichiometry factor of Pt : CO = 1 : 1. Pretreatment was done at 250 °C under H_2_ flow (50 sccm) for 2 hours. 10% CO gas balanced with He was used as the gas pulse, and the measurement was carried out at 50 °C.

### 2.3 Performance of the CO oxidation reaction

CO oxidation was carried out in a flow reactor as described elsewhere.^25^ Initially, 50 mg of the catalyst was loaded into a tubular reactor. Before the reaction, the catalyst was reduced at 250 °C under flowing H_2_ (5% H_2_ in He, 45 ml min^−1^) for 2 hours, and then cooled to room temperature. Product CO_2_ gas was not detected in the reactor before the reaction started. The reactant gas composition was 4% CO, 10% O_2_, and 86% He (carrier gas). The total gas flow rate was 50 ml min^−1^, controlled by mass flow controllers (BROOKS instrument). The space velocity of the reaction was 15 000 h^−1^. CO oxidation was carried out until reaching 100% CO conversion (at temperatures between 60 and 200 °C). The gas mixture passing through the catalyst powder was analysed using gas chromatography (DS science). The turnover frequency (TOF) was calculated based on the active Pt sites measured from the chemisorption of CO. The stability of the catalysts was measured in 5 consecutive heating–cooling cycles. After the first CO oxidation test, the catalysts were cooled down to room temperature for 1 hour under He gas before commencing the next reaction cycle.

## 3 Results and discussion

### 3.1 Characterization of the synthesized Fe-doped ZnO samples

**Fig. 1:**
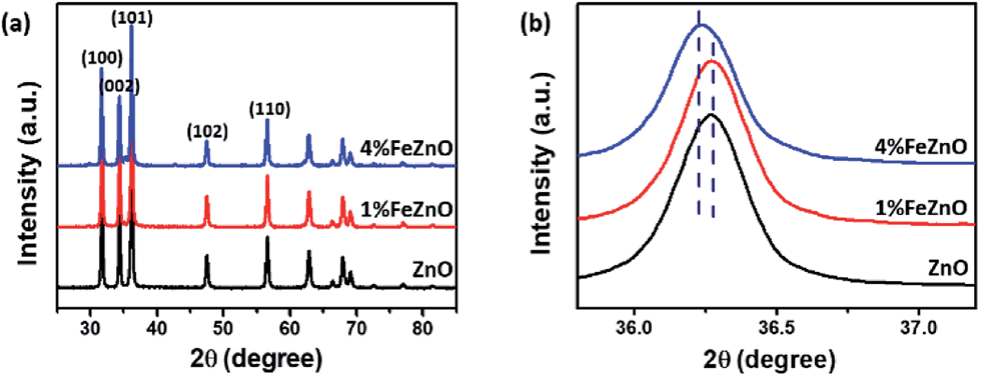
(a) XRD patterns of the synthesized *x*%FeZnO samples. (b) The (101) diffraction peaks that show a shift between the samples.

The crystalline structure of the synthesized *x*%FeZnO samples was investigated using the XRD technique. All the samples exhibited high crystalline hexagonal wurtzite belonging to the *C*^4^_6*v*_ space groups (*P*6_3_*mc*) of ZnO (JPCDS card no. 36-1451) without detecting other crystalline phases or impurities (Fig. 1a). The ionic replacement of the dopant into the lattice of the host material results in a slight distortion of the unit cell, which leads to a small shift of all the strong diffraction peaks in the XRD patterns. From the magnified (101) diffraction peaks for all the samples (Fig. 1b), the peak for the 1%FeZnO sample did not show a considerable shift compared with that for ZnO, which was probably caused by the very small Fe dopant loading. However, the peak shift towards the lower 2*θ* angle for the 4%FeZnO sample compared with the other two was easily distinguished. The shift can be attributed to the compressive stress of the unit cell in the crystal structure when ions with larger radii were used as the dopant. Because the ionic radii of iron and zinc are different (Fe^2+^: 0.074 nm, Fe^3+^: 0.064 nm, and Zn^2+^: 0.072 nm),^26^ it is likely that Fe^2+^ ions were substituted into the Zn site. A similar observation has been interpreted from the incorporation of Fe dopant ions into the lattice of ZnO host structures.^27–29^ The data obtained from XRD indicated that the Fe dopant did not form crystalline segregated bi-phases, but was well-incorporated into the ZnO crystal lattice. Higher Fe loading can result in the formation of ZnFe_2_O_4_ and Fe_2_O_3_ phases.^30^ Because the focus of this work is to study the influence of metal doping on catalytic activity for CO oxidation, other crystalline structures than Fe-doped ZnO should not exist. Hence, we limited the Fe loading to 4% to prevent the emergence of new crystalline phases in the samples.

**Fig. 2:**
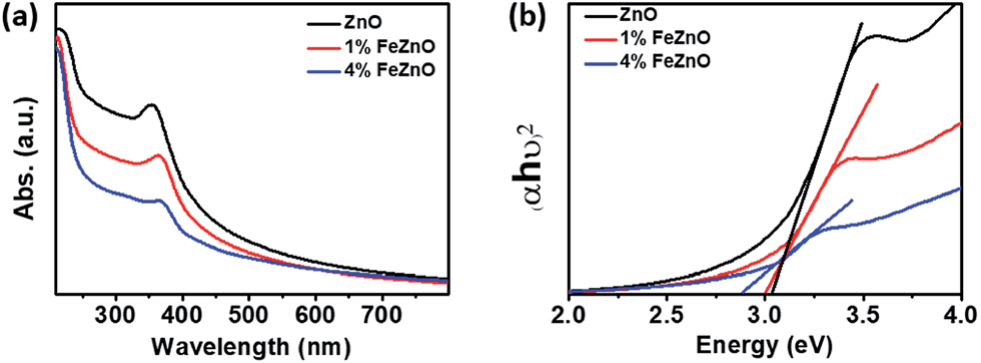
(a) UV-vis absorption spectra and (b) Tauc's plot for optical band gap estimation of the *x*%FeZnO samples.

The optical band gap of the materials was estimated using UV-vis spectroscopy based on the absorption peak centered at around 370 nm in each sample, as seen in Fig. 2a. The data in Fig. 2b, which shows Tauc's plot for band gap estimation, exhibited a downward trend as the amount of Fe doping increased (see Table 1). This is consistent with the gradual change in colour from the light white of ZnO to the orange of 4%FeZnO (ESI Fig. S1†) and is explained by the formation of defect states in the band gap by the transfer of 3d electrons from the trivalent dopants (*e.g.*, Fe^3+^, Al^3+^, Ga^3+^) to the conduction band of the n-type semiconductors.^21,31^ The UV-vis data provide further evidence supporting the successful doping of Fe into the ZnO.

**Table 1:** Actual iron loadings, physicochemical properties, and band gap estimates of the synthesized *x*%FeZnO

Sample notation	Fe loading (wt%)	Surface area (m^2^ g^−1^)	Average pore size (nm)	Band gap estimate
ZnO	0.00	11.90	16.40	3.04
1%FeZnO	1.09	13.00	18.90	3.00
4%FeZnO	3.81	14.30	16.00	2.92

The texture properties of the *x*%FeZnO samples were assessed using N_2_ adsorption–desorption analysis (see Table 1). The ZnO sample exhibited the lowest surface area, whereas loading small amounts of Fe (*i.e.*, 1 and 4%) showed a negligible difference in both the surface area and average pore size compared with the ZnO sample. The surface areas of all the samples were relatively small, with the highest at 14.3 m^2^ g^−1^ for the 4%FeZnO sample. Table 1 also provides the actual Fe loading of the synthesized *x*%FeZnO, as determined using ICP-OES analysis. The actual Fe loadings were about 1% and 4%, which are in good agreement with the calculated values.

### 3.2 Characterization of the synthesized Pt/*x*%FeZnO catalysts

**Fig. 3:**
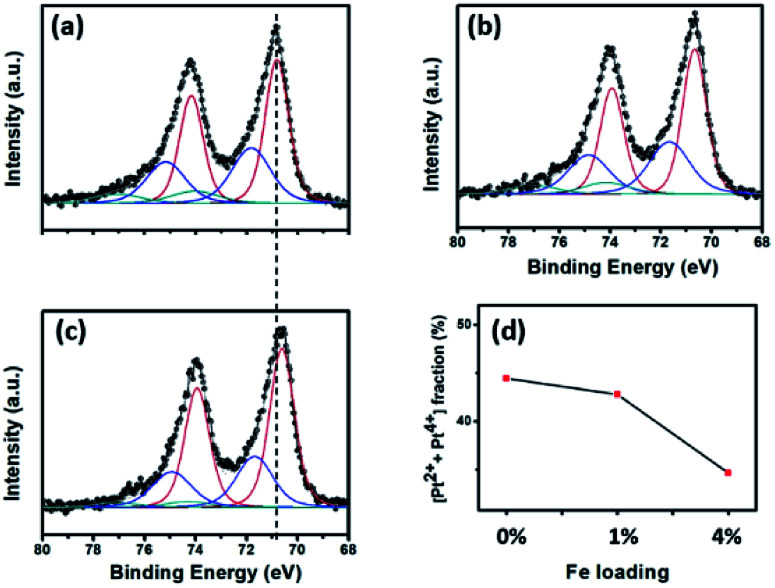
XPS analysis of the Pt 4f core level for the (a) Pt/ZnO, (b) Pt/1%FeZnO, (c) Pt/4%FeZnO catalysts; the dashed line shows the peak shift between the Pt/ZnO and Pt/4%FeZnO samples. (d) A comparison of the oxide fraction of Pt in the catalysts as a function of Fe loading.

The real amount of Pt loading (see Table 2) was measured using ICP-OES analysis. In general, the Pt loading was similar in all the samples, implying the insignificant role of this factor when comparing the catalytic activity. XPS analysis was performed to investigate the surface oxidation states of the Pt NPs in each sample. All the Pt peaks were calibrated based on adjusting the C 1s peak position to 284.8 eV. Fig. 3a–c shows the XPS spectra after fitting using the CasaXPS program. In all the samples, the Pt 4f peaks were composed of both the metallic (Pt^0^) and oxidation (Pt^2+^ and Pt^4+^) states;^32^ however, the former was predominant, which was most likely because of the use of the strong reductant NaBH_4_ in the preparation step. It is worth noting that all the Pt 4f peaks for the Pt/4%FeZnO sample shifted to a lower binding energy (*i.e.*, ∼0.2 eV) in comparison with those for the Pt/ZnO catalyst, indicating that the surface of the Pt NPs in the Pt/4%FeZnO catalyst had a higher electron density because of electron transfer from the support. Moreover, Fig. 3d exhibited a downward trend for the oxide fraction of the Pt with the increase in Fe dopant loading, namely from 44.4% in the Pt/ZnO catalyst to 34.7% in the Pt/4%FeZnO catalyst, which is consistent with the conclusion inferred from the observed peak shift. Data obtained from the XPS analysis suggest that 4%FeZnO has the strongest interaction with the Pt NPs. The electronic states of the Fe and Zn were also analysed to provide more details about the charge transfer phenomenon. XPS spectra of the Fe 2p and Zn 2p core levels for all the samples were collected and shown in ESI Fig. S3.† The Fe 2p peaks in ESI Fig. S3a† showed very low intensity because of the small amount of Fe existing on the surface of the FeZnO support. It should be noted that only small Fe loadings, namely, 1% and 4% were introduced to the ZnO. Moreover, because Fe was well-doped into ZnO lattice, a major fraction of the Fe is located deep inside the bulk. XPS analysis, which can only detect the elemental composition of the surface of substances (about several atomic layers), therefore, showed very low-intensity Fe 2p peaks. On the other hand, the Zn 2p core level of all the samples, as seen in ESI Fig. S3b,† did not show any peak shift, which was most likely because the Zn 2p spectra reflected the electronic status of the Zn^2+^ ions in the ZnO lattice, which were predominant in comparison with those existing at the interface between the FeZnO support and the Pt NPs.

**Table 2:** Characterization and catalytic activity of the synthesized Pt/*x*%FeZnO catalysts

Samples	Pt wt% (ICP-OES)	*D*_Pt_ (nm)	Pt dispersion (%)		*T*_100_ (°C)	TOF (s^−1^) at 126 °C	*E*_a_ (kcal mol^−1^)
			Before reaction	After reaction			
Pt/ZnO	2.29	2.20	3.22	1.83	147	0.60	13.00
Pt/1%FeZnO	2.08	2.50	3.21	1.86	138	1.34	11.80
Pt/4%FeZnO	2.04	2.40	3.48	3.01	126	5.37	8.66

**Fig. 4:**
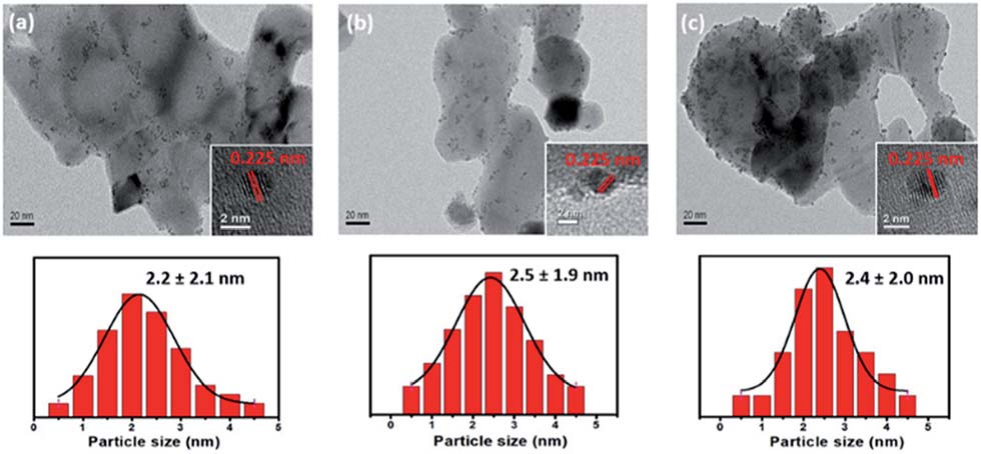
TEM images of the synthesized (a) Pt/ZnO, (b) Pt/1%FeZnO, (c) Pt/4%FeZnO catalysts and their corresponding particle size distributions.

Fig. 4 shows TEM images of the synthesized Pt/*x*%FeZnO catalysts and their corresponding particle size distributions. The Pt NPs can be observed as small dark spots, while the catalyst supports are brighter. The inset HR-TEM images show a random Pt NP on each sample. The lattice fringe was 0.225 nm in all cases, which is attributed to the Pt (111) surface (JCPDS card no. 65-2868). To get additional evidence of the Pt deposition, EDS mapping of a random area on the Pt/4%FeZnO sample, which is representative of the whole, was carried out. As seen in ESI Fig. S2,† the EDS mapping data clearly confirm the existence of Pt. Both the TEM and EDS data ascertained the successful deposition of Pt NPs on the synthesized *x*%FeZnO supports. From the EDS mapping, it is worth noting that a homogeneous distribution of Zn and Fe could also be observed across the entire mapped area, which further supported the successful doping of Fe into the ZnO. A statistic survey of 200 particles was performed to evaluate the size distribution of the Pt NPs in each sample. The average Pt NP diameters (*D*_Pt_) were 2.2, 2.5, and 2.4 nm for 0, 1, and 4% Fe loading, respectively (see Table 2). Although the mean sizes of the Pt NPs in all the catalysts were similar, we can see from the TEM images that the metal NPs were well dispersed on the 4%FeZnO support; while on the ZnO, the NPs migrated close to one another to form local domains with high NP density. The difference in dispersion behaviour for the Pt NPs could be explained by the strength of the interaction between the Pt NPs and the supports. When preparing catalysts using the deposition–precipitation method, the hydroxide form of the Pt was initially anchored on the surface of the support. During the reduction process to induce the Pt NPs, there is a tendency for the Pt NPs to agglomerate to minimize surface energy. When the interaction between the Pt NPs and surface of the support was strong enough to overcome the agglomeration force, the Pt NPs could grow well dispersed across the surface. With a somewhat weaker interaction between the Pt NPs and the surface of the support, agglomeration of the Pt NPs could occur during NP growth, but quite slowly. Due to the relatively low rate of agglomeration in comparison with that of reduction, the Pt NPs would still have similar sizes as in the abovementioned case, but they assembled closer to each other to form local domains with high NP density, as seen in the Pt/ZnO catalyst. Otherwise, if this interaction was very weak or depleted, the Pt NPs would aggregate to become larger NPs. From the dispersion behaviour of the Pt NPs on each support, we assume that there is a stronger interaction between the Pt NPs and the 4% Fe loading sample compared with the other samples. To confirm this assumption, Pt dispersion for all the synthesized catalysts before and after the CO oxidation reaction was evaluated using the CO chemisorption method (see Table 2). While Pt dispersion on the Pt/4%FeZnO sample decreased slightly after the reaction from 3.48 to 3.01%, Pt dispersion on the other samples dropped significantly. This agrees well with the TEM images of the catalysts after CO oxidation (ESI Fig. S4†), which exhibited sintering of the Pt NPs to form larger NPs in the Pt/ZnO and Pt/1%FeZnO samples. Combined with the XPS data, the TEM observations and the chemisorption data clearly indicate that Pt NPs have the strongest interaction with the 4%FeZnO support, thus resulting in the best NP sintering resistance.

### 3.3 CO oxidation of the synthesized Pt/*x*%FeZnO catalysts

**Fig. 5:**
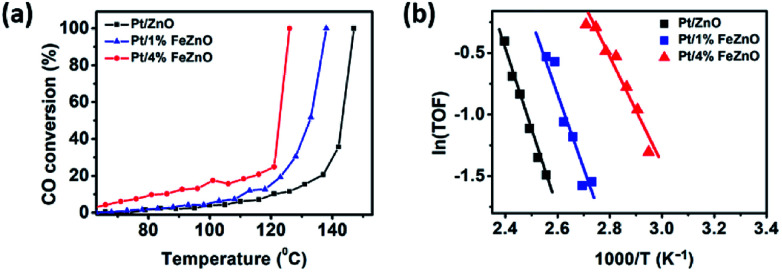
(a) CO conversion as a function of temperature and (b) Arrhenius plot for the synthesized Pt/*x*%FeZnO catalysts.

**Fig. 6:**
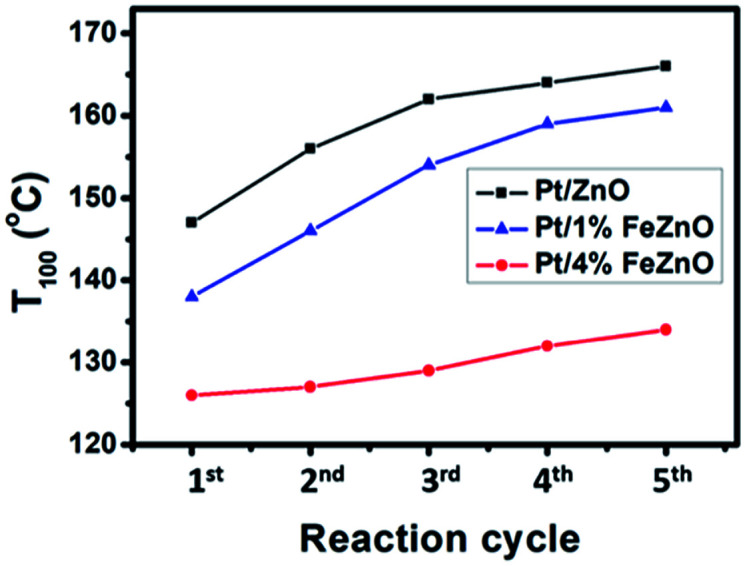
Stability test for the synthesized Pt/*x*%FeZnO catalysts during the CO oxidation reaction.

The dependence of CO conversion on the reaction temperature for all the synthesized catalysts is depicted in Fig. 5a. Generally, CO conversion increases with higher reaction temperatures. The temperatures corresponding to 100% CO conversion (*T*_100_) decreased with higher Fe dopant loading. These temperatures were 147, 138, and 126 °C with for 0, 1, and 4% Fe loading, respectively (see Table 2). The TOF of the synthesized catalysts, based on the Pt chemisorption analysis and calculated at 126 °C, increased significantly with higher Fe dopant loading, namely 0.60, 1.34, and 5.37 s^−1^ for 0, 1, and 4% Fe loading, respectively. The activation energies (*E*_a_) of the CO oxidation reaction for the synthesized Pt-based catalysts were calculated from the Arrhenius plot (Fig. 5b). The 4% Fe loading catalyst had the smallest *E*_a_ value of 8.66 kcal mol^−1^, while the *E*_a_ values for the Pt/ZnO and Pt/1%FeZnO catalyst were 13.0 and 11.8 kcal mol^−1^, respectively. From the abovementioned results, it was concluded that the catalytic activity for the CO oxidation reaction increases with increased Fe loading. Because long-term durability is among the most vital factors for evaluating catalysts, we also investigated the stability of the synthesized catalysts by performing the reaction for five consecutive heating–cooling cycles. The Pt/4%FeZnO catalyst, which had the advantage of higher catalytic activity, also exhibited better stability compared with the others. The *T*_100_ for this sample only increased slightly, whereas the *T*_100_ for the other two catalysts rose dramatically during the five consecutive cycles (Fig. 6).

The electronic and geometric effects are plausible explanations for the improved catalytic activity and stability. In our study, the enhanced activity and stability of the Pt/*x*%FeZnO catalysts could be attributed to the presence of the Fe dopant (as confirmed by XRD results), which improved electron transfer between the Pt NPs and the support. Enhancement of electron transfer between the metal and the support indicates a stronger metal–support interaction, which could stabilize the NPs and prevent them from sintering.^22^ As a result, the Pt/4%FeZnO catalyst, which had the strongest interaction between the Pt NPs and the support, exhibited the highest sintering resistance, as is evident from the TEM image (ESI Fig. S4†) and the chemisorption data after the reaction, which thus promotes both the catalytic activity and stability. Metal particle sintering has been an intrinsic problem for improving catalyst performance and stability.^33,34^ Particularly, the detrimental effect of Pt sintering on the catalytic CO oxidation reaction has been reported elsewhere. For instance, in comparison with a Pt/SiO_2_ catalyst calcined at 600 °C, Jung *et al.* observed a decrease in catalytic activity for the CO oxidation reaction when the catalyst was calcined at 750 °C.^25^ Accordingly, the sintering effect that deactivated the Pt NPs was the main reason for the drop in catalytic performance. Yang *et al.* systematically investigated the link between sintering in a Pt/Al_2_O_3_ catalyst and its activity for CO oxidation.^35^ They found an apparent relation between the degree of metal sintering and the loss of catalytic activity. In the current work, sintering to form larger Pt NPs is assumed to occur at high temperature under the oxidative conditions over the course of the reaction resulting from a weak metal–support interaction. A study of Pt size evolution using an *in situ* TEM technique with the CO oxidation reaction over a Pt/ZnO catalyst would be able to provide a direct observation of this sintering process. Nevertheless, the work reported here may provide inspiration for the use of a metal dopant as an efficient approach for designing metal–oxide support catalysts to achieve better catalytic performance.

## 4 Conclusions

A series of Fe-doped ZnO materials with different Fe loading (*i.e.*, 0, 1, and 4%) were synthesized *via* the co-precipitation method followed by calcination. Pt NPs were deposited on the *x*%FeZnO samples using deposition–precipitation to fabricate catalysts for the CO oxidation reaction. Characterization of all the samples using TEM, XRD, EDS, and UV-vis indicated that the Fe ions were well-doped into the ZnO lattice and that the Pt NPs were successfully grown on the supports. For the CO oxidation reaction, we found that the catalytic activity of the synthesized catalysts was enhanced with increased Fe loading, with the highest TOF value of 5.37 s^−1^ at 126 °C from the Pt/4%FeZnO catalyst. The increased Fe doping promoted the interaction between the Pt NPs and the support, leading to a higher resistance for metal sintering, which resulted in the increase in catalytic activity.

## Conflicts of interest

There are no conflicts to declare.

## Supplementary Material

RA-008-C8RA03664K-s001
