# Supplementary material for: Iron-doped ZnO as a support for Pt-based catalysts to improve activity and stability: enhancement of metal–support interaction by the doping effect
Source: RSC Adv. 2018 Jun 12;8(38):21528–33. doi: 10.1039/c8ra03664k (PMC9080930; doi:10.1039/c8ra03664k)
Supplement: RA-008-C8RA03664K-s001 [file RA-008-C8RA03664K-s001.pdf]

Electronic Supplementary Material (ESI) for RSC Advances.
This journal is © The Royal Society of Chemistry 2018

Electronic Supplemental Information (ESI)

Iron-doped ZnO as Support for Pt-based Catalysts to Improve Activity and Stability: Enhancement of Metal–Support Interaction by the Doping Effect

Si Bui Trung Tran[a], Han Seul Choi[a,b], Sun Young Oh[a,b], Song Yi Moon[a,b], Jeong Young Park[*,a,b]

[a] Center for Nanomaterials and Chemical Reactions, Institute of Basic Science (IBS), Daejeon 305-701, Republic of Korea

[b] Graduate School of EEWS and Department of Chemistry, Korea Advanced Institute of Science and Technology (KAIST), Daejeon 305-701, Republic of Korea

* E-mail: jeongypark@kaist.ac.kr

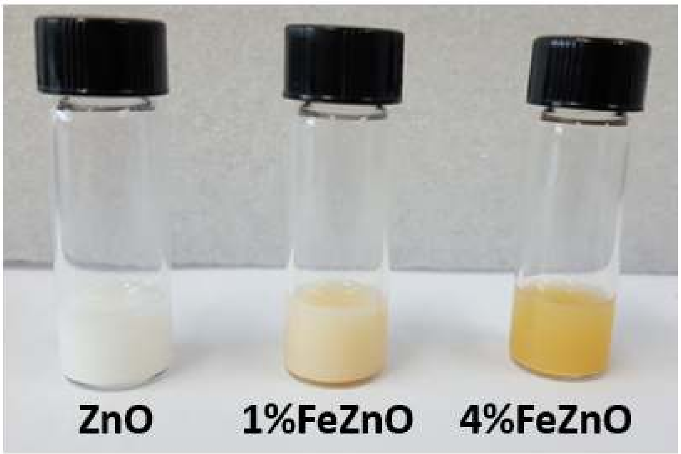

Fig. S1 Photograph of the samples.

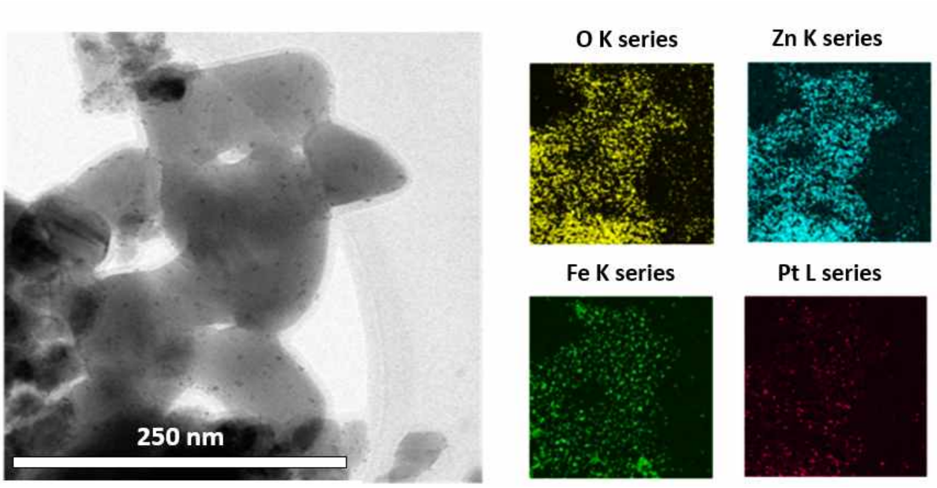

Fig. S2 (left) SEM image and (right) corresponding EDS mapping of the Pt/4%FeZnO sample.

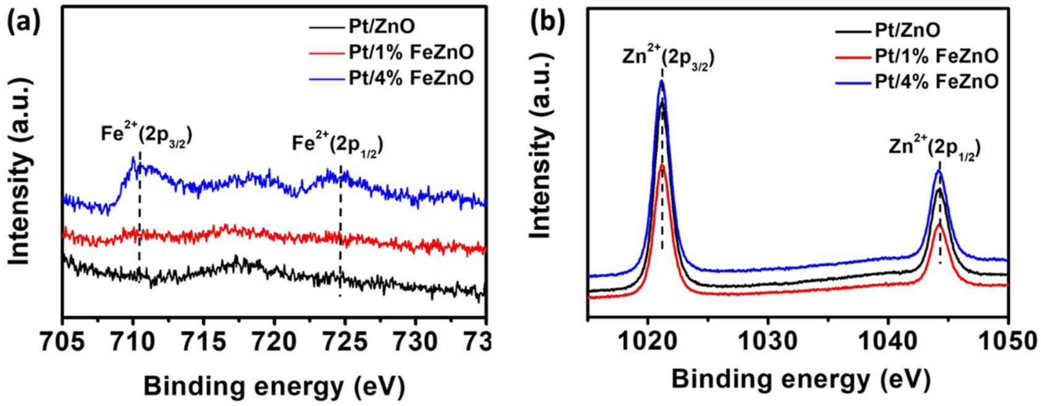

Fig. S3 XPS spectra of (a) Fe 2p and (b) Zn 2p core levels for the catalysts Pt/ZnO, Pt/1%FeZnO, and Pt/4%FeZnO.

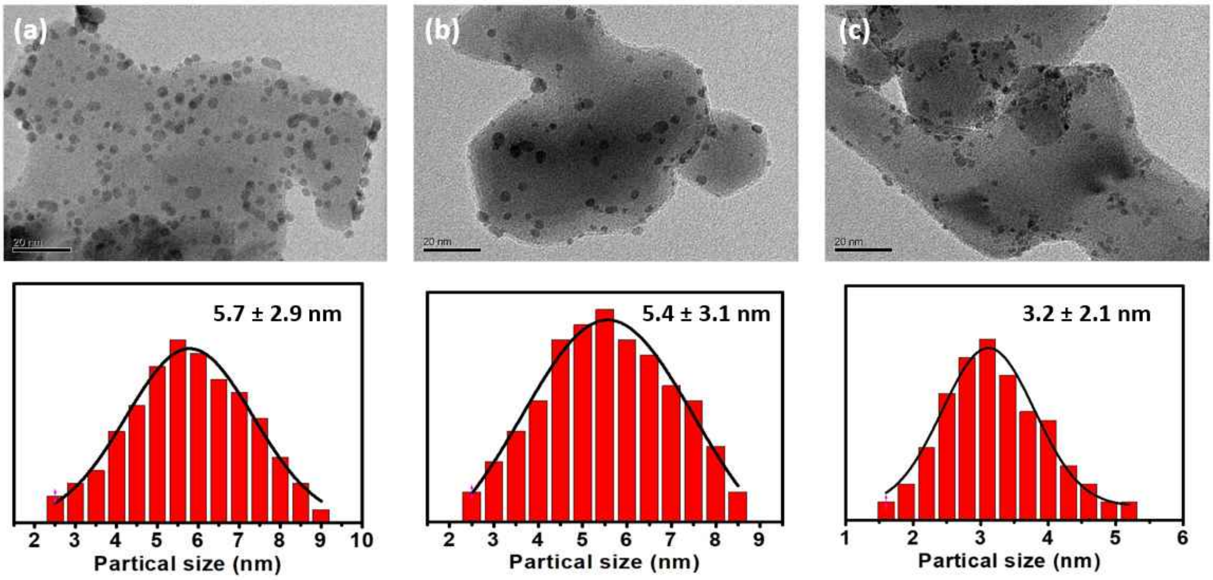

Fig. S4 TEM images of the (a) Pt/ZnO, (b) Pt/1%FeZnO, and (c) Pt/4%FeZnO samples collected after the CO oxidation reaction and their corresponding particle size distributions
